# Willingness and Barriers to Undertaking Cardiopulmonary Resuscitation Reported by Medical Students after the SARS-CoV-2 Pandemic—Single-Center Study

**DOI:** 10.3390/jcm13020438

**Published:** 2024-01-13

**Authors:** Filip Jaskiewicz, Wojciech Timler, Jakub Panasiuk, Katarzyna Starosta, Marcin Cierniak, Remigiusz Kozlowski, Monika Borzuchowska, Klaudiusz Nadolny, Dariusz Timler

**Affiliations:** 1 Emergency Medicine and Disaster Medicine Department, Medical University of Lodz, 90-419 Lodz, Poland; jakub.panasiuk@umed.lodz.pl (J.P.); katarzyna.starosta@umed.lodz.pl (K.S.); marcin.cierniak@umed.lodz.pl (M.C.); dariusz.timler@umed.lodz.pl (D.T.); 2 Department of Family Medicine, Medical University of Lodz, 90-419 Lodz, Poland; wojciech.timler@umed.lodz.pl; 3 Department of Management and Logistics in Healthcare, Medical University of Lodz, 90-419 Lodz, Poland; remigiusz.kozlowski@umed.lodz.pl (R.K.); monika.borzuchowska@umed.lodz.pl (M.B.); 4 Department of Emergency Medical Service, Faculty of Medicine, Silesian Academy in Katowice, 40-555 Katowice, Poland; prrm.knadolny@interia.pl

**Keywords:** out-of-hospital cardiac arrest, cardiopulmonary resuscitation, CPR, resuscitation, education

## Abstract

Most of the studies in the field of willingness and barriers to resuscitation (CPR) were conducted before the SARS-CoV-2 pandemic. The aim of the study was to assess the number and types of barriers to CPR among medical students after the pandemic ended. This study was based on a survey. The data was collected from 12 April 2022 to 25 May 2022. A total of 509 complete questionnaires were obtained. The number of barriers depending on the time elapsed from the last CPR course did not differ significantly (Me = 4 [IQR 2–6] vs. Me = 5 [IQR 3–7]; *p* = 0.054, respectively). The number of all barriers reported by respondents differed significantly and was higher in those reporting fear of coronavirus (Me = 4 [IQR 2–6] vs. Me = 7 [IQR 4–9]; *p* < 0.001, respectively). A total of 12 out of all 23 barriers were significantly more frequent in this group of respondents. Barriers to CPR are still common among medical students, even despite a high rate of CPR training. The pandemic significantly affected both the number and frequency of barriers. The group of strangers and children, as potential cardiac arrest victims, deserve special attention. Efforts should be made to minimize the potentially modifiable barriers.

## 1. Introduction

Sudden cardiac arrest (SCA) is a serious problem for public health around the world [1,2,3,4]. Despite the efforts made in the last three decades and recent progress, the survival rate in an out-of-hospital cardiac arrest (OHCA) is still very low in many countries [1,2,3,4,5,6]. Even lower is the survival rate with a good neurological outcome. Early, high-quality cardiopulmonary resuscitation (CPR) and early use of the automatic external defibrillator (AED) are the key factors for successful resuscitation in OHCA [3,4]. It is currently known that such measures may be associated with up to a three-fold increase in survival rate and, extremely importantly, a favorable neurological outcome [2,3,4,5,6,7]. For this reason, actions have been undertaken around the world to implement resuscitation guidelines more effectively. While until recently, research and training processes were focused only on practical skills, increasing attention is now focused on the human factor. The 2021 guidelines of the European Resuscitation Council (ERC) emphasize that the main objective of resuscitation training should be to increase the frequency of CPR in OHCA [8]. The key to achieving this effect is to increase the readiness of witnesses (bystanders) to initiate CPR. For this reason, the factors affecting this readiness have now become the subject of many studies [8,9,10,11,12,13,14,15,16,17].

In parallel, many research papers assess the presence of potential barriers to CPR [16,17,18,19,20,21,22,23]. Extremely importantly, the results indicate their large diversity both in terms of nature and their potential modifiability, with a positive impact of earlier resuscitation training on a reduced number of barriers being one of the most common positive factors [7,8,9,10,11,12]. Importantly, a literature review showed that most of the studies in this field had been conducted before the outbreak of the SARS-CoV-2 pandemic, which could have had a significant impact on the perceived barriers to providing CPR by potential bystanders [24,25]. The essence of the issue prompted us to identify barriers that prevent delivering CPR to potential OHCA victims as reported by first-year medical students and analyze the impact of the SARS-CoV-2 pandemic on their characteristics. This particular academic year of future medical doctors analyzed in the current study differs from all previous ones due to the experience of the lockdown period and the reality of the pandemic. Actions taken to identify the number and types of potential barriers in particular populations may, in the future, help to better organize educational programs, improve methods for conducting CPR training, and develop other activities focused on achieving the key goal, i.e., increasing the frequency of bystander CPR in out-of-hospital cardiac arrest.

## 2. Aim

The general aim of the study was to identify and assess the number and types of barriers to CPR among first-year medical students in the period after the end of the SARS-CoV-2 pandemic.

Specific aims were also set to evaluate the impact on the number and occurrence of different types of barriers in the context of:

- past CPR training history;
- declared fear related to the outbreak of the SARS-CoV-2 pandemic.

## 3. Materials and Methods

### 3.1. Study Setting

This study was based on an online survey questionnaire developed by the authors. The data was collected from 12 April 2022 to 25 May 2022 among first-year medical students and students with a specialization in dentistry. Information about the possibility of participating in the study, together with a link to the questionnaire, was sent to the participants’ e-mail addresses twice (on the first day of the study and 3 days before the planned end of the study). The questionnaire made it possible for the participants to submit their answers only once. The study was approved by the Bioethics Committee at the Medical University of Lodz (number RNN/77/22/KE).

### 3.2. Participants

The research population consisted of first-year medical students and students with a dental specialization at the University of Medical Sciences in Lodz. The total number of possible study participants was 687. Participation in the study was voluntary and anonymous. Willingness to participate in the study and a returned, completed questionnaire were the inclusion criteria. Lack of consent to participate in the study, graduation from another medical faculty, or practice of another medical profession were the exclusion criteria. No information on whether the participants were vaccinated before participating in the study was recorded. This variable was not taken into account, as in Poland, medical students are not legally obliged to be vaccinated against the SARS-CoV-2 virus.

### 3.3. Data Sources/Measurement

In this study, the size of the test was calculated using G*Power 3.1. In reference to the number of all first-year medicine students from the recruitment of the study group in Poland (10,371), at least 450 respondents needed a level of confidence of α = 0.97 and a maximum error of 0.05. Due to the voluntary nature of the study and potential exclusion criteria, it was assumed that the questionnaire would be presented to a minimum of 550 first-year medical students at the Medical University of Lodz.

An online survey questionnaire (Supplementary File S1) was used as the research tool to collect the data. The process of its development and evaluation was as follows:

Stage 1: Literature review to identify questions used in similar research, including literature reviews, original papers, guidelines, and international recommendations.
Stage 2: Analysis of questions used in other researchers’ questionnaires and their critical verification in relation to consistency with the aims of the presented study.
Stage 3: Preparation of a list of questions appropriate to verify the chosen aims of the study.
Stage 4: Content evaluation by experienced subject matter experts (*n* = 4): removal of three questions divergent from the study aims.
Stage 5: Presentation of the questionnaire in a pilot form to 45 respondents to evaluate whether:
  - the content of the questions is understandable to the respondents;
  - closed questions provide at least one answer choice that would apply to every respondent;
  - questions are interpreted in the same way by all the respondents;
  - the questionnaire creates a positive impression and motivates respondents to answer the questions;
  - any aspect of the questionnaire suggests any bias.
Stage 6: Minor corrections and completing the questionnaire evaluation process.

### 3.4. Statistical Analysis

Statistical analyses were performed using PQStat 1.8.4.136. Quantitative variables are presented using basic descriptive statistics: the arithmetic mean (x), standard deviation (SD), median (Me), interquartile range [IQR], and percentages (%). The distribution of answers to questions was analyzed with the Chi-squared test or the Fisher’s exact test (if the Cochran’s condition was not met) and calculated measures of dependence—C-Pearson (adjusted) and Phi coefficients. The number of barriers depending on the time of participation in the first aid course and the fear of delivering CPR due to the SARS-CoV-2 pandemic was compared using the Mann–Whitney U test. The test probability at the level of *p* < 0.05 was considered significant, and the test probability at the level of *p* < 0.01 was considered highly significant.

## 4. Results

A total of 531 out of 687 first-year medical students at the Medical University in Lodz took part in this anonymous and voluntary study. As a result, 509 complete questionnaires meeting the inclusion criteria (22 were rejected due to other medical education or performing other medical professions) were obtained. The study group came from 15 of the 16 total provinces in Poland. Among the respondents, there were also 2 (0.4%) students from Belarus and 2 students from Ukraine. The mean age of respondents was 20 ± 2.1 years (Me = 20; min = 18; max = 40). Men accounted for 65%, and 0.8% of respondents refused to state their gender. The characteristics of respondents in terms of past CPR training are presented in Table 1.

All study participants declared participation in some form of resuscitation training in the past (at school/at a driving school/at work/other form of training), including over half in the last year. In practice, the majority were trained to assess consciousness and the presence of normal breath and perform CPR on an adult manikin. However, just over half of the respondents had hands-on training in the use of AED or resuscitation on a child manikin (Table 1). Despite the high incidence of a lack of previous hands-on training to use AED (44.6% of respondents), 85.3% of the study group respondents declared its use if the device was nearby.

The analysis of answers to the question about the declared willingness to assess consciousness and breath as well as deliver CPR to various SCA victims showed the greatest readiness of the study participants to act in the case of a family member or a person they know. Respondents would be less likely to help a child or a stranger with a case of cardiac arrest (Table 2). A statistically significant relationship was found between past hands-on training on a child manikin and readiness to perform resuscitation for this type of victim. Respondents who had trained on a pediatric manikin in the past were more likely to undertake child resuscitation than those who had not (91.9 vs. 84.7%, respectively; *p* = 0.011).

The time elapsed since participating in the CPR course did not significantly affect the willingness to deliver CPR to any of the types of victims (Table 2).

Also, the number of barriers depending on the time elapsed from the last CPR course did not differ significantly (Me = 4 [IQR 2–6] vs. Me = 5 [IQR 3–7]; *p* = 0.054, respectively). The incidence of barriers in the total study group is presented in Table 3. Each respondent could indicate any number of barriers, with Me = 4 [IQR 3–7] declared barriers for the total study group.

For 19 out of all 23 assessed barriers, no statistically significant relationships were found between their occurrence and the duration of the resuscitation course. However, the frequency of four barriers reported by respondents depended on the time elapsed since the course (Table 4), and it was higher in all four cases in the group of students trained > 1 year before the survey.

The second specific aim of the present study was to evaluate respondents’ readiness to assess consciousness, breath, and deliver CPR to SCA victims with various characteristics, depending on the reported fear following the outbreak of the SARS-CoV-2 pandemic. In this case, respondents confirming fear related to the pandemic were statistically significantly less likely to deliver CPR to children and strangers (Table 5).

The number and frequency of individual barriers were also analyzed, depending on whether a given respondent reported that the outbreak of the SARS-CoV-2 pandemic contributed to their concerns about performing CPR. In this case, the number of all barriers reported by respondents differed highly statistically and was higher in those reporting fear of coronavirus (Me = 4 [IQR 2–6] vs. Me = 7 [IQR 4–9]; *p* < 0.001, respectively). Additionally, in the case of this variable, the analysis showed that 12 out of all 23 barriers were significantly more frequent in this group of respondents than in the case of those who did not report fear related to the outbreak of the pandemic (Table 6). Also, the percentage of each of these 12 barriers was higher in this group compared to all respondents in the study group.

## 5. Discussion

SCA has been a major public health problem for years, both in terms of the persistently high mortality rates and the costs of organizing health care and rehabilitation for survivors [1,2,5]. An early call for medical help, the initiation of high-quality resuscitation by a bystander, and performing automatic external defibrillation if an AED is available on site are the keys to increasing the chances of survival with a good neurological outcome in OHCA. From this perspective, the role of a bystander is invaluable. Statistics show that a high percentage of OHCA cases occur in the presence of another person [3,4,6]. However, the percentage of resuscitation attempts undertaken by witnesses remains too low in many countries [6].

Research to identify factors influencing both the willingness to initiate and potential barriers to providing CPR by bystanders is not new [16,20,21]. Unfortunately, as the literature reviews indicate, the methodology of individual studies, the characteristics of respondents, and the scope and nomenclature of barriers vary greatly. In practice, this makes it difficult to identify reasons why bystanders might not initiate CPR. This, in turn, prevents researchers and educators from finding ways to eliminate them. Additionally, more recent results and conclusions drawn from many years of research and analysis could potentially be disturbed by the global SARS-CoV-2 pandemic [18,24,25].

The selection of the study group is not accidental, as this generation was affected by a phenomenon that had not occurred on a global scale for several decades before starting university studies and choosing a profession. A huge advantage of identifying barriers in this population at such an early stage is the potential possibility of modifying them in the later years of study at the University of Medical Science in Lodz. Unfortunately, it may not be possible to implement this type of action in relation to the entire population or even its individual groups with different characteristics than the present study participants. The literature review conducted for this study identified 23 barriers, the potential importance of which was reported by other authors. For the specific purposes of the study, previous participation in CPR training was selected as one of the factors potentially influencing the occurrence of barriers. This is also the result of a literature review and the attention paid to this aspect by many previous publications. Unfortunately, in this case too, there are large discrepancies in the reports regarding the training history of the surveyed respondents. Therefore, in the presented study, a criterion based on the Guidelines of the European Resuscitation Council was used, and the respondents were divided into two groups based on their previous training history, i.e., those who participated in training < 12 months and >12 months before the start of the study. Reports from other authors also indicate that the method of training and its nature (online without hands-on training, hybrid—online with hands-on training, or traditional hands-on, instructor-led training) may have a different impact on the readiness and willingness to start resuscitation among bystanders. For this reason, the survey questionnaire specified the issue of practical training in assessing consciousness and recognizing the presence of normal breathing, delivering CPR on an adult manikin, a child manikin, and hands-on AED training for a more detailed understanding of the general concept of training history analyzed in the present study.

The impact of the declared fear of some respondents caused by the outbreak of the SARS-CoV-2 pandemic was the second factor analyzed that may have potentially influenced barriers to CPR. Currently, there are few reports on the impact of the pandemic on potential bystanders who would start CPR, but it is unquestionable and consistent with Suggestions for Change and Areas for Future Research, the recently published Scientific Statement from the American Heart Association, that such an exceptional situation is a factor whose impact should be taken into account when considering the topic [26].

### 5.1. Study Group as a Whole

The results of answers to questions about the history of resuscitation training may seem extremely satisfactory due to the fact that all study participants reported that they had been trained at least once [19,27,28,29,30]. Additionally, the fact that more than half of them (51.9%) had been trained in the last 12 months is also very optimistic. This is surprisingly high compared to the reports from other studies. However, it should be kept in mind that the present study was conducted with a very specific group of participants. Firstly, the age of the respondents indicates that, according to the law in force in Poland, some of them, after reaching the age of eighteen, could already participate in a driving license course during which CPR classes are obligatory. The second important factor may be the fact that, according to the core curriculum of the Ministry of Education in Poland, every high school student has CPR in the program (Subject—Safety Education). Additionally, this group of respondents may not correspond to the entire population, which means that their interests in the medical field could also result in more frequent exposure to resuscitation training.

The results on the practical form of training received by respondents in the past are also very optimistic. Almost all of them received hands-on training to assess consciousness and normal breathing, as well as perform chest compressions on an adult manikin (95.7 and 94.5%, respectively). Much more disturbing, however, is the fact that such a small part of the study group has ever had the opportunity to practice resuscitation on a child manikin or to learn how to use an automatic defibrillator in practice (53.1 and 55.4%, respectively). This result is surprising, especially when considering the secondary school curriculum. Both in Poland and in many countries, the costs of conducting such training during school classes are accepted, and training equipment enabling the training of at least several hundred students does not pose a great financial challenge [31]. The cost of a child manikin enabling real-time feedback, or the training version of an automatic defibrillator, is no more than $300. Such disturbing results in relation to the lack of pediatric CPR training and key training in the chain of survival of an adult with cardiac arrest and the use of a defibrillator may result from decision-making and organizational errors. Especially since a detailed analysis showed that respondents who had trained on a pediatric manikin in the past were more likely to declare a potential undertaking of child resuscitation than those who had not. This issue certainly requires a more careful analysis.

The declarations of the total study group regarding their readiness to deliver CPR to a family member and a known person were very high. However, in the case of strangers and children potentially requiring resuscitation, the willingness to perform CPR reported by the respondents was much lower. However, in relation to all types of victims, the results are higher than those in other studies conducted in various populations or an analysis conducted among nearly a thousand working adults in Poland at the turn of 2019 and 2020 [27,32,33].

In relation to the barriers reported in the total study group, three of them occurred in approximately 1 out of every 2 respondents: vomit on the victim’s face, uncertainty of cardiac arrest recognition, and fear of doing harm. While the presence of vomit on the victim may be an unmodifiable barrier for some bystanders, the other two are highly disturbing. The lack of certainty in the diagnosis of SCA in a population that performed so well, as shown by the results of the training history and practical training, is alarming. This may mean that the problem of recognizing the symptoms of SCA, including gasping, is not discussed thoroughly enough during CPR education [3,5,8]. This barrier should be considered crucial in both psychological and practical contexts. It may be a strong reason for the bystander not to start CRP [26,34]. Similarly disturbing is the frequent fear of harming an OHCA victim. It may also indicate shortcomings in both the way CPR education is carried out and the training programs themselves. The Suggestions for Change and Areas for Future Research from the American Heart Association place great emphasis on this issue. At key points, it emphasizes that training should clearly demonstrate how the benefits of a potential rescue outweigh the risk of harm to an unresponsive patient [26]. Similarly disturbing is the high incidence (approximately 1 out of every 3 respondents) of barriers that could be potentially modifiable during CPR training, i.e., lack of confidence to initiate CPR, insufficient CPR skills, and fear of legal consequences. Their occurrence in the study group with such high training rates may also indicate the need for a more in-depth analysis of the impact of the details and quality of CPR training on the occurrence of barriers [8,26,34]. Future research should take this issue into strong consideration.

### 5.2. The Impact of CPR Training History

The main goal of the present study was to identify barriers that may occur during an attempt to perform CPR among young people who have chosen to become medical doctors. Interestingly, the analysis of the impact of the time spent completing a CPR course showed no statistically significant differences in relation to any type of victim. The recent International Liaison Committee on Resuscitation (ILCOR) Recommendations [35] indicate a negative correlation between readiness to perform CPR and time elapsed since initial CPR training (over 3–12 months). Other authors report that resuscitation training in general has a significant impact on the willingness to perform CPR. In their literature reviews, Scapigliati et al. and González-Salvado et al. highlighted the role of repeated training and community initiatives [36,37]. However, as mentioned earlier, the definition of training and the time elapsed since the course itself vary depending on the publication.

Also, in relation to the number of reported barriers to CPR, respondents who trained in the last year and those who completed a course more than 12 months before do not differ significantly. However, a more detailed analysis showed that 4 of the 23 assessed barriers were significantly more common in the group of respondents who received CPR training > 1 year before the study. It is worth paying attention to the specificity of these four barriers: fear of doing harm, fear of legal consequences, insufficient CPR knowledge, and a child victim. The more frequent occurrence of the first two, as discussed earlier, may indicate paying too little attention to these important issues during CPR courses. As can be seen from the present study, these dangerous and unjustified barriers increase as time passes after training. The third barrier related to the loss of knowledge in the field of CPR over time seems natural and consistent with other studies [35,36,37]. The fear of resuscitating a child in the total study group may be related to the low frequency of hands-on training using a pediatric manikin in the past. In this type of victim, the fear of doing harm, which increases with time after training, may also have a strong impact and correlation [35].

### 5.3. The Impact of the Reported Fear of the SARS-CoV-2 Pandemic

As for the second assessed variable, i.e., fear related to the recent pandemic, respondents who reported this factor were statistically significantly less likely to deliver CPR to a stranger or a child. It is worth noting that in this group of respondents, the lowest percentages of declared readiness to perform life-saving activities were recorded for these two types of victims. While the fear of a disease transmitted by droplets and in the form of an aerosol may obviously be associated with a reduced willingness to resuscitate strangers, such poor readiness to start resuscitation in the case of a pediatric SCA victim can be considered at least intriguing. It is worth noting that this difference was, however, much smaller than in the case of strangers (8.3 vs. 28.5%, respectively). All the more so because the fear related to the resuscitation of a child did not appear in the group of 12 out of all 23 barriers shown to be more common among respondents who reported fear related to the pandemic. In this group of respondents, there were statistically significantly more barriers declared. Apart from “vomit on the victim’s face,” which again topped the list, fear of an unspecified infectious disease was the second most commonly declared barrier in this group. Again, the very frequent occurrence of the previously discussed barriers may be of great concern: uncertainty of cardiac arrest recognition, fear of doing harm, lack of confidence to initiate action, insufficient CPR skills, and fear of legal consequences, which seem to dominate regardless of the variable factor. The fear of blood on the victim, which was also much more common than in the total study group and the subgroup denying fear related to the pandemic, may be related to the awareness of the risk of infection associated with contact with this particular bodily fluid.

It is not known whether respondents mentioning the SARS-CoV-2 pandemic were characterized by a generally higher degree of fear in relation to other issues or whether, in real life, this issue would be limited only to CPR situations. It is clear, however, that although the time elapsed since CPR training did not significantly affect the willingness to initiate CPR in any type of victim, and a detailed analysis showed a statistical difference for only four barriers, the results are significantly different in the case of respondents reporting fear related to the SARS-CoV-2 pandemic. Although research on the relationship between pandemic-related fear and barriers reported from the perspective of potential resuscitation is new, the presented findings seem to be consistent with the reports on the decrease in the frequency of CPR due to the pandemic and its consequences [13,24,25]. Once again, following the Scientific Statement from the American Heart Association, such an exceptional situation is a factor whose impact should be taken into account and requires more detailed analysis in future research.

### 5.4. Challenges and Possible Future Directions

**Figure 1:**
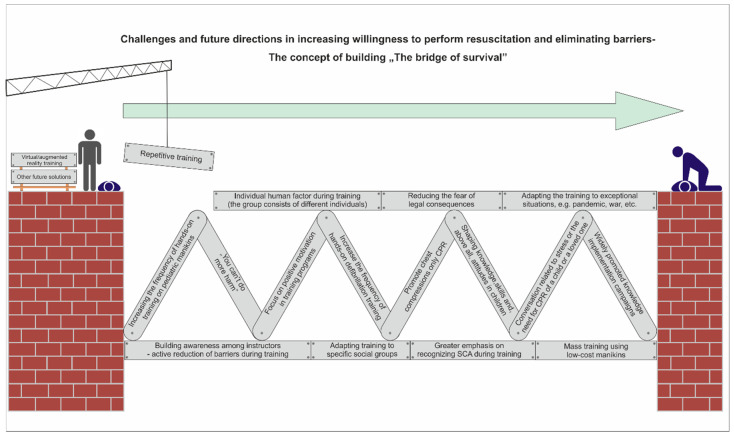
The concept of continuous construction of “the bridge of survival”.

In order to create a modifiable point of reference for future research regarding the search for factors influencing the initiation of resuscitation by bystanders and thus reducing barriers causing lack of action, it is worth making an attempt to present a new concept. The concept of continuous construction of “the bridge of survival” (Figure 1). Its essence would be to build a bridge allowing a person witnessing SCA to move to the point where he or she is able to undertake effective resuscitation based on prior recognition of cardiac arrest. This bridge concept is built of bricks that are the result of scientific research and recommendations [7,8,9,10,11,12,13,16,17,18,19,20,21,22,23,24,33,34,35,36,37,38,39]. These bricks may have a different quality and may be replaced or rebuilt as new variables appear over time. This means a completely new approach, different from the previous ones. They assume the need for constant verification and modification guided by reality. This approach is to be largely driven not only by new scientific achievements but, above all, by the variability of the human factor and the reality in which the victim and bystanders live and function. This first outline of the concept is based on a literature review, the results of the present study, and the authors’ previous research. However, it is very important to realize that this is not a target presentation. The concept itself, by its nature, should generate more questions than answers and become the basis for researchers’ investigations and continuous work on the reorganization of many factors. Regardless of the fact that it is largely based on a literature review, in relation to the current study and its results, it may constitute a proposal for adaptation among first-year medical students in Poland during the pandemic period. As its important feature is the possibility of reconstruction, the final variant of the bridge should be based on further research on different populations of bystanders. 

## 6. Limitations

This study had several limitations. The main ones are the inability to directly transfer the conclusions from the study to first-year medical students in other countries and education systems. This is due to both systemic differences and the course of the pandemic itself in individual regions of the world. Additionally, the impact of information on previous participation in first aid training may be different for each participant because the form of this training, its duration, and its program, apart from the details included in the survey, may have been different. Finally, the current study assessed the impact of selected factors only on respondents’ declarations. Their consistency with actual performance in a real-life situation may vary. No information on whether the participants were vaccinated before participating in the study was recorded. This variable was not taken into account, as in Poland, medical students are not legally obliged to be vaccinated against the SARS-CoV-2 virus, but it may potentially affect the results obtained. Finally, the results of the current study cannot be directly transferred to the entire population of bystanders. In future studies, identifying and analyzing barriers and factors influencing the willingness to start resuscitation should take into account the specificity of a given population.

## 7. Conclusions

Barriers to CPR are still common, even among first-year medical students with a high rate of CPR training. The exceptional situation associated with the SARS-CoV-2 pandemic significantly affected both the number and frequency of barriers. The group of strangers and children, as potential cardiac arrest victims, deserve special attention and action in the future. During CPR training for medical students in Poland, efforts should be made to increase the willingness to deliver CPR and minimize the potentially modifiable barriers such as uncertainty in the diagnosis of cardiac arrest, fear of harming the victim, concern over legal consequences, or poor self-assessment of one’s own skills.

## Figures and Tables

**Table 1:** Respondent characteristics in terms of past CPR training.

*n* = 509		*n*	%
Participation in a first aid course in the past	YES, <1 year	264	51.9
	YES, >1 year	245	48.1
Hands-on training to assess consciousness and normal breathing in the past		487	95.7
Hands-on CPR training on an adult manikin in the past		481	94.5
Hands-on CPR training on a child manikin in the past		270	53.1
Practical training in AED use in the past		282	55.4
Declaration of AED use if it was available nearby?		434	85.3

**Table 2:** Comparison of the number of participants who declared willingness to undertake CPR on individual victims depending on previous training.

Type of Victim	Study Group *n* = 509 (%)	CPR Course < 1 Year *n* = 264 (%)	CPR Course > 1 Year *n* = 245 (%)	*p*-Value
Family member	503 (98.8)	243 (99.2)	260 (98.5)	0.687
Person you know	500 (98.2)	242 (98.8)	258 (97.7)	0.506
Child	451 (88.6)	213 (86.9)	238 (90.2)	0.254
Stranger	401 78.8)	723 (77.6)	883 (79.9)	0.512

**Table 3:** The incidence of barriers declared by all study participants.

Declared Barriers	*n* = 509	%
Vomit on the victim’s face	270	53.1
Uncertainty of cardiac arrest recognition	249	48.9
Fear of doing harm	247	48.5
Fear of an unspecified infectious disease	190	37.3
Lack of confidence to initiate action	174	34.1
Insufficient CPR skills	156	30.6
Anxiety over the stress reaction/panic	147	28.8
Fear of legal consequences	142	27.9
Perceptible odor of alcohol	129	25.3
Fear of getting infected with SARS-CoV-2	126	24.8
Blood on the victim	113	22.2
Insufficient CPR knowledge	101	19.8
Inability to perform CPR due to physical malfunction/health	91	17.8
A child victim	90	17.6
Many other witnesses at the scene of the event	87	17.0
Low socio-economic status of the victim	84	16.5
No particular reason	31	6.0
Victim lying face to the ground	30	5.8
Advanced age of the victim	28	5.5
Victim lying on a bed	13	2.5
Victim in a sitting position	12	2.3
A woman is the victim	9	1.7
A man is the victim	6	1.1

**Table 4:** The presence of barriers depending on CPR training history.

Declared Barriers	CPR Course < 1 Year	CPR Course > 1 Year	*p* Value
	*n* = 264 (%)	*n* = 245 (%)	
Fear of doing harm	116 (43.9)	131 (53.5)	*p* = 0.031
Fear of legal consequences	60 (22.7)	82 (33.4)	*p* < 0.001
Insufficient CPR knowledge	40 (15.2)	61 (24.9)	*p* = 0.005
A child victim	36 (13.6)	54 (22.1)	*p* = 0.013

**Table 5:** Comparison of the number of participants declaring their readiness to deliver CPR to individual victims depending on reported fear of the SARS-CoV-2 pandemic.

Type of Victim	Study Group *n* = 509 (%)	Fear of Performing CPR Declared as Caused by the SARS-CoV-2 Pandemic		*p*-Value
		NO	YES	
		*n* = 383 (%)	*n* = 126 (%)	
Family member	503 (98.8)	378 (98.7)	124 (98.5)	0.818
Person you know	500 (98.2)	377 (98.3)	123 (97.7)	0.552
Child	451 (88.6)	346 (90.3)	103 (82.0)	0.024
Stranger	401 (78.8)	329 (85.9)	72 (57.4)	< 0.001

**Table 6:** The occurrence of barriers depending on the declaration of fear associated with the SARS-CoV-2 pandemic.

Declared Barriers	Fear of Providing CPR Declared as Caused by the SARS-CoV-2 Pandemic		*p* Value
	NO	YES	
	*n* = 383 (%)	*n* = 126 (%)	
Vomit on the victim’s face	182 (47.5)	88 (69.8)	*p* < 0.001
Fear of an unspecified infectious disease	109 (28.5)	81 (64.3)	*p* < 0.001
Uncertainty of cardiac arrest recognition	171 (44.6)	78 (61.9)	*p* < 0.001
Fear of doing harm	173 (45.1)	74 (58.7)	*p* = 0.008
Lack of confidence to initiate CRP	119 (31.1)	55 (43.6)	*p* = 0.009
Insufficient CPR skills	104 (27.2)	52 (41.3)	*p* = 0.002
Blood on the victim	62 (16.2)	51 (40.5)	*p* < 0.001
Fear of legal consequences	95 (24.8)	47 (37.3)	*p* = 0.006
Perceptible odor of alcohol	85 (22.2)	44 (34.9)	*p* = 0.004
Insufficient CPR knowledge	66 (17.3)	35 (27.8)	*p* = 0.010
Inability to perform CPR	59 (15.4)	32 (25.4)	*p* = 0.011
Many other witnesses at the scene of the event	58 (15.2)	29 (23.0)	*p* = 0.041

## Data Availability

The digital data supporting this study are curated by Filip Jaskiewicz and available on a reasonable request.

## Supplementary Materials

The following supporting information can be downloaded at: https://www.mdpi.com/article/10.3390/jcm13020438/s1, File S1: Survey Questionnaire Form; File S2. The concept of continuous construction of “The bridge of survival”.
