# Supplementary material for: Willingness and Barriers to Undertaking Cardiopulmonary Resuscitation Reported by Medical Students after the SARS-CoV-2 Pandemic—Single-Center Study
Source: J Clin Med. 2024 Jan 13;13(2):438. doi: 10.3390/jcm13020438 (PMC10816474; doi:10.3390/jcm13020438)
Supplement: Supplementary file 1 [file jcm-13-00438-s001.zip › jcm-2758749-supplementary.pdf]

File S1: Survey Questionnaire Form

Barriers in undertaking cardiopulmonary resuscitation reported by medical students - the state of knowledge in the period after the SARS CoV-2 pandemic

1. Age in years

Enter your answer

2. Gender

    Male
    Woman
    I don't want to answer that question

3. Direction

    Medical
    Medical - Dental Department

4. Voivodship (origin)

Enter your answer

5. Have you attended a first aid course or school activity (at school, driving school, university classes, privately, at work)?

    NO, never (in which case go to question 10)
    YES, within 1 year
    YES, > 1 year

6. Have you trained the skills of assessing consciousness and the presence of normal breathing (on a phantom or a phantom)?

    YES
    NO

7. Have you trained compressions and rescue breaths on an adult phantom during the training or classes?

    YES
    NO

8. Have you trained compressions and rescue breaths on a child's phantom during the training or classes?

    YES
    NO

9. Have you trained in the use of an automated defibrillator (AED) during your training or classes?

    YES
    NO

10. Does the outbreak of the SARS CoV2 pandemic make you afraid of performing CPR?

    YES
    NO

11. Which of the following would you undertake a consciousness assessment, breathing assessment and CPR? (select all that apply)

    Family member
    Child
    A person I know (acquaintance, co-workers, neighbors, etc.)

Stranger
I would not take any action, regardless of the circumstances and who the victim is

12. Would you use an automated defibrillator (AED) if it was available nearby?
YES
NO

13. Tick all the statements that define your barriers/fear of starting CPR:
I don't know enough to perform CPR
I don't have enough skills to perform CPR
I don't have the confidence to start CPR
I'm afraid of being infected with unspecified disease
I'm afraid of being infected with coronavirus
I'm afraid I'm going to panic
I'm afraid I'm going to hurt the victim
I'm afraid of the legal consequences of incorrect actions
I'm afraid that the victim will be in a sitting position
I'm afraid that the victim will be lying on the bed
I'm afraid that the victim will be lying on his stomach
I'm afraid the victim will be covered in blood
I'm afraid that the victim's will be covered in vomit
I'm afraid that there will be a odour of alcohol coming from the victim
I'm afraid of CPR if the victim is a woman
I'm afraid of CPR if the victim is a man
I'm afraid I won't be able to do it because of my physical condition
Low socio-economic status of the victim
Multiple witnesses to the incident at the scene
Uncertainty about whether I will correctly diagnose cardiac arrest
The victim's advanced age
The victim is a child
For no particular reason
